# Monoubiquitination of EEA1 regulates endosome fusion and trafficking

**DOI:** 10.1186/2045-3701-3-24

**Published:** 2013-05-23

**Authors:** Harish N Ramanathan, Guofeng Zhang, Yihong Ye

**Affiliations:** 1 Laboratory of Molecular Biology, National Institute of Diabetes and Digestive and Kidney Diseases, National Institutes of Health, 5 center drive, Bethesda, MD 20892, USA; 2 Biomedical Engineering and Physical Science Shared Resource, NIBIB, Bethesda, MD 20892, USA; 3 Current address: Section of Microbial Pathogenesis, Yale University School of Medicine, 295 Congress Ave., New Haven, CT 06536, USA

**Keywords:** EEA1, Endosome recycling, Ubiquitin, Endosome fusion, Rab5, E3-independent ubiquitination

## Abstract

**Background:**

Early endosomal autoantigen 1 (EEA1) is a membrane tethering factor required for the fusion and maturation of early endosomes in endocytosis. How the activity of EEA1 is regulated in cells is unclear.

**Results:**

Here we show that endogenous EEA1 is prone to monoubiquitination at multiple sites, owing to an intrinsic affinity to ubiquitin conjugating enzymes (E2). The E2 interactions enable a ubiquitin ligase (E3) independent mechanism that decorate EEA1 with multiple mono-ubiquitin moieties. Expression of an ubiquitin-EEA1 chimera that mimics native mono-ubiquitinated EEA1 generates giant endosomes abutting the nucleus. Several lines of evidence suggest that this phenotype is due to increased endosome fusion and a simultaneous blockade on an endosome recycling pathway. The latter is likely caused by diminished endosome fission in cells expressing ubiquitin-EEA1.

**Conclusion:**

Our results demonstrate that ubiquitination may dramatically affect the activity of an endosome fusion factor to alter endosome morphology and trafficking pattern, and thereby implicating an unexpected role of ubiquitin signaling in endocytosis.

## 

The survival of eukaryotic cells depends on intake of nutrients from the environment. This process is at least in part dependent on endocytosis, a cellular pathway in which a portion of the plasma membranes invaginate, forming vesicular structures termed endosomes. Endocytosis also imparts regulatory functions in a large number of processes ranging from cellular signaling transduction to protein quality control [1,2]. Upon entering the cytosol, the early endosomes undergo a complex maturation process as they traverse towards a perinuclear region via the cellular cytoskeletal system. Meanwhile, through acquisition of new proteins and lipids as well as by fusing with other endosome vesicles, early endosomes mature into late endosomes, which eventually fuse with the lysosomes to turn over internalized molecules [3]. It is also known that early endosomes constantly undergo a fission process [4]. Some fission products form recycling endosomes, which help to bring certain internalized membrane receptors back to the plasma membrane for re-usage [5,6]. In addition to receiving materials from the plasma membranes, the endosomes can also exchange contents with the Golgi apparatus through vesicular trafficking. Thus, the endocytic membrane system is highly dynamic and interactive, a feature required to maintain membrane equilibrium and structural integrity of many intracellular organelles.

Early endosomal autoantigen 1 (EEA1) is an essential component of the endosomal fusion machinery [7,8]. It contains a C-terminal FYVE domain responsible for binding phosphatidyl inositol-3-phosphate on the membrane, two binding sites that impart communications with the master endocytosis regulator Rab5, and a long helical domain required for oligomerization of EEA1 [7,9-13]. These features confer membrane binding and fusion-inducing activities to EEA1 because once the membrane-associated EEA1 oligomerizes, it tethers endosomes and primes them for subsequent fusion [14].

We previously identified EEA1 as a potential substrate for the ubiquitin selective segregase p97 [15], an ATPase that operates in many cellular processes to separate ubiquitinated polypeptides from membranes, DNA, or large protein complexes [16,17]. Our results suggested that p97 may regulate endosome fusion by controlling the oligomerization status and therefore the tethering activity of EEA1. In this study, we further demonstrate that similar to other p97 substrates, EEA1 is subject to regulation in cells by ubiquitination, which appears to control not only the size of the endosomes, but also their trafficking pattern.

## Results

### Monoubiquitination of EEA1 in cells

**Figure 1:**
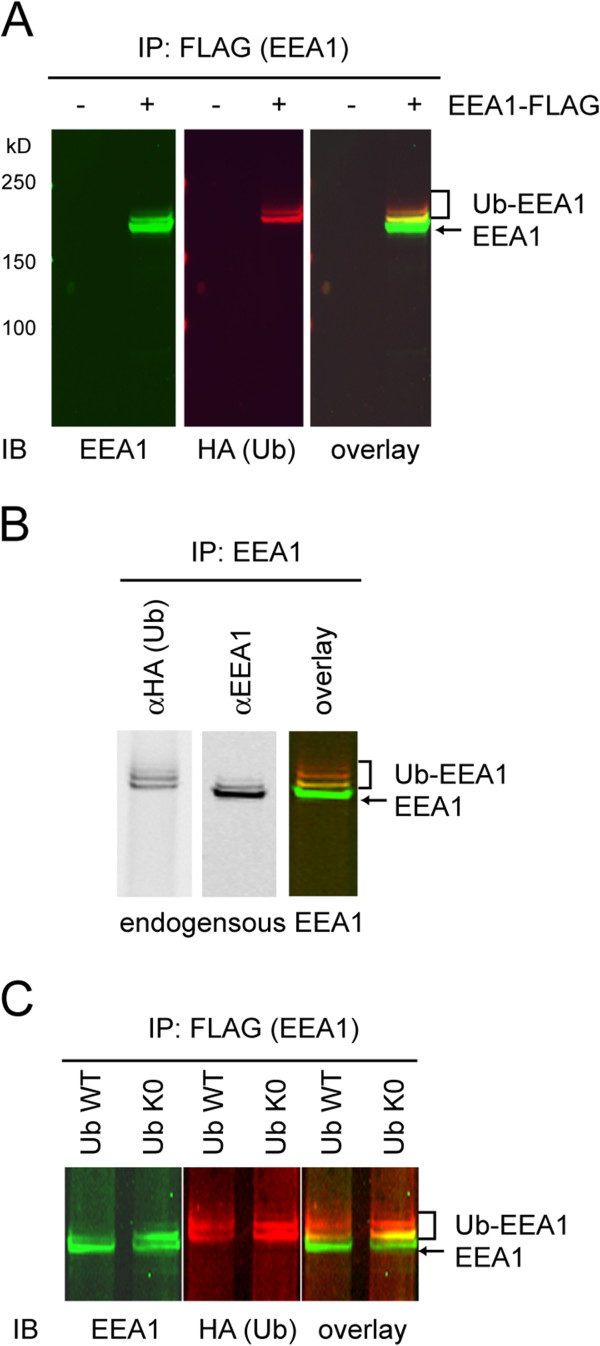
**EEA1 undergoes mono-ubiquitination in cells. A**, Cell extracts prepared from HEK293 cells transfected with HA-tagged ubiquitin- (HA-Ub) and EEA1-FLAG-expressing plasmids (+) or a control empty vector (−) were subject to immunoprecipitation with FLAG antibodies. The precipitated materials were analyzed by immunoblotting (IB) with the indicated antibodies. The bracket indicates ubiquitinated EEA1 species. **B,** As in **A**, except that cells expressing HA-Ub were used and that immunoprecipitation was performed with anti-EEA1 antibodies. **C,** As in **B**, except that cells expressing the indicated Ub variants were used.

To investigate whether EEA1 is ubiquitinated in cells, we expressed full-length FLAG-tagged wild type EEA1 together with hemagglutinin (HA)-tagged ubiquitin in HEK293 cells. EEA1-FLAG appeared functional as a fraction of it was localized to the endosomes [15]. Cell extracts were subject to immunoprecipitation with the FLAG antibody. Immunoblotting showed that when EEA1-FLAG was expressed, the EEA1 antibody stained not only the ectopically expressed EEA1, but also a few EEA1-containing bands migrating more slowly. These additional EEA1-containing species corresponded to ubiquitinated EEA1 because they were also recognized by anti-HA antibodies, as demonstrated by two-color immunoblotting (Figure 1A). As expected, no ubiquitinated EEA1 could be detected when FLAG-EEA1 plasmid was omitted during transfection. Ubiquitinated EEA1 was also detected with endogenous EEA1 using a similar method (Figure 1B). The migration pattern of ubiquitinated EEA1 remained similar when a ubiquitin mutant lacking lysine residues was expressed (Figure 1C). Since this lysine-less ubiquitin mutant did not support the formation of ubiquitin chain, our results suggest that a fraction of EEA1 is ubiquitinated in cells at several sites, each with a single ubiquitin moiety.

### EEA1 has an intrinsic affinity to ubiquitin conjugating enzymes

Protein ubiquitination usually requires three types of enzymes, an E1 activating enzyme, an E2 conjugating enzyme and an E3 ligase [18]. A conjugation reaction also depends on ubiquitin and ATP. In most cases, these enzymes would form polymerized chains on substrates and the type of ubiquitin chains can dictate the functional consequence of a ubiquitination reaction [19].

**Figure 2:**
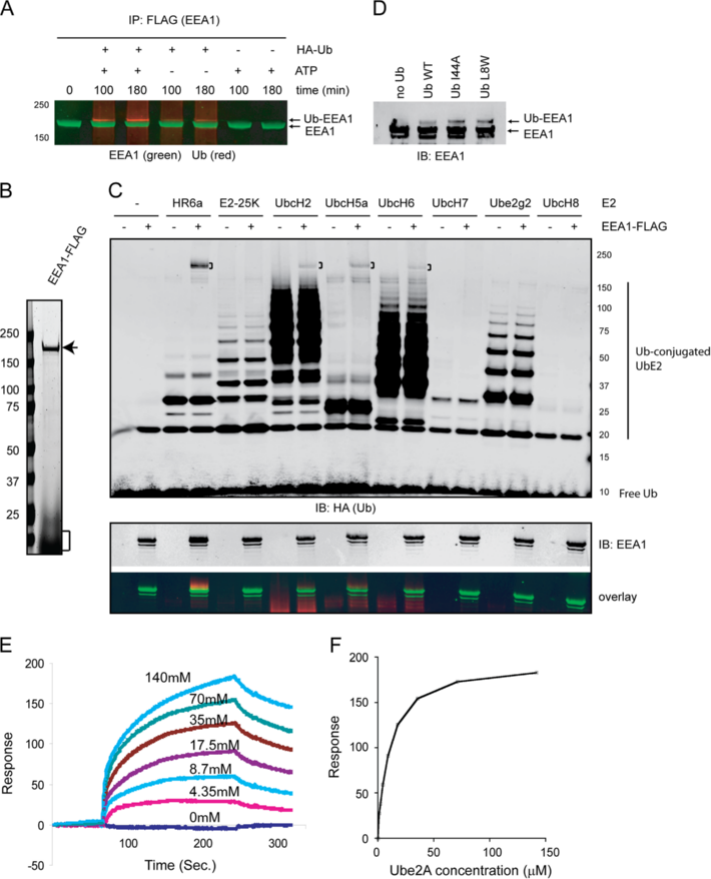
**EEA1 can interact with E2 to undergo E3-independent mono-ubiquitination. A**, In vitro ubiquitination was performed with a post-nuclear extract isolated from cells expressing EEA1-FLAG in the presence of HA-tagged ubiquitin and ATP as indicated. The reactions were analyzed by dual color immunoblotting with the indicated antibodies. **B**, A Coomassie-blue stained gel showing the purified EEA1 (indicated by the arrow). The bracket indicates 3XFLAG peptide. **C**, Purified EEA1 was incubated with the indicated E2 ubiquitin conjugating enzymes together with E1, HA-Ub, and ATP. Where indicated, EEA1-FLAG was omitted as negative controls. The reactions were analyzed by immunoblotting with the HA (top panel) and EEA1 antibodies (bottom panel). **D**, As in A, except that the indicated ubiquitin variants were used. **E**, **F**, Interaction of EEA1 with Ube2A. **E**, Purified Ube2A was injected into a CM5 chip immobilized with EEA1-FLAG at the indicated concentrations. The interaction of Ube2A with EEA1 was monitored by Surface Plasmon Resonance. **F**, The interaction of EEA1-FLAG with Ube2A was plotted against the E2 concentrations.

To dissect the mechanism by which EEA1 preferentially undergoes monoubiquitination, we developed an *in vitro* ubiquitination assay using post-nuclear extract. To this end, cells expressing EEA1-FLAG were treated with a low salt buffer to disrupt the plasma membrane. We reasoned that the enzymes involved in EEA1 ubiquitination likely reside in either cytosol or on the endosome membranes. We therefore obtained a post-nuclear extract fraction containing both endosomes and cytosol and incubated it with HA-tagged ubiquitin and an ATP regenerating system. Immunoblotting showed that EEA1 was rapidly ubiquitinated in the presence of ATP and ubiquitin (Figure 2A). In the absence of exogenously added ATP, only a small amount of ubiquitinated EEA1 was generated, likely due to the residual endogenous ATP, which was present at millimolar concentrations in cells. By contrast, no ubiquitinated EEA1 was detected in the absence of ubiquitin. This was not surprising given that endogenous ubiquitin was significantly diluted during cell lysis.

We next wished to reconstitute ubiquitination of EEA1 using purified proteins. We incubated purified EEA1 (Figure 2B), ubiquitin activating enzyme (E1), ubiquitin, and ATP together with a panel of E2 conjugating enzymes. After incubation, immunoblotting showed that several E2 enzymes were able to support EEA1 ubiquitination even in the absence of any E3 ubiquitin ligases. These included Ube2A(HR6a), Ube2H(UbcH2), Ube2D1(UbcH5a) and Ube2E1(UbcH6). Ube2A appeared to be the most efficient one in mediating EEA1 ubiquitination (Figure 2C).

As mentioned above, ubiquitination often involves an E3 ligase that serves as a matchmaker to bring a substrate in proximity to a ubiquitin-charged E2 enzyme [20]. One method that allows bypass of an E3 ligase in ubiquitination is to utilize a ubiquitin recognition motif (UIM) in a substrate, which binds directly to the ubiquitin moiety on an activated E2 [21,22]. This mechanism requires a hydrophobic surface composed of Ile44 and Leu8 in ubiquitin, which serves as the docking site for UIM. EEA1 does not contain any identifiable UIMs, but it might have a cryptic ubiquitin binding site that mediates its own ubiquitination. To exclude this possibility, we performed in vitro EEA1 ubiquitination using ubiquitin mutants defective in UIM binding [23]. The result showed that these ubiquitin mutants were as effective as wild type ubiquitin in ubiquitination of EEA1 (Figure 2D). This observation raised the possibility that EEA1 may directly communicate with an E2 enzyme(s) to receive ubiquitin from it. This conclusion was indeed confirmed by Surface Plasmon Resonance (SPR) experiments using purified EEA1 and an E2. These in vitro binding experiments demonstrated that EEA1 had an intrinsic affinity to Ube2A with Kd of ~9.7 ± 0.54 μM (Figure 2E, F). By contrast, Ube2G2 bound EEA1 with a significantly reduced affinity (data not shown), consistent with the fact that Ube2G2 could not ubiquitinate EEA1 *in vitro*.

### Expression of a ubiquitin-EEA1 chimera generates giant vacuole-like endosomes

**Figure 3:**
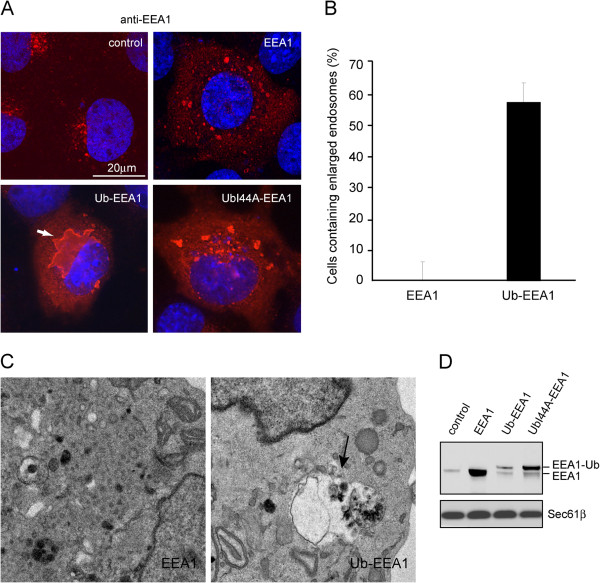
**Ub-EEA1 expression induced vacuole-like endocytic structures. A**, COS7 cells transfected with the indicated plasmids were immunostained with anti-EEA1 antibodies in red. Cells were counter-stained with DAPI to reveal the nuclei. **B**, Quantification of Ub-EEA1-induced giant vacuole phenotype. The percentage of cells with enlarged vacuole-like endosomes in either EEA1 or Ub-EEA1 expressing cells is indicated. Error bars, SD (n = 3). **C**, EEA1- or Ub-EEA1-expressing cells were fixed and analyzed by transmission electron microscopy. The arrow indicates a representative vacuole-like structure in Ub-EEA1-expressing cells. **D**, Extracts prepared from cells transfected with the indicated plasmids were analyzed by immunoblotting to determine the expression levels of the indicated proteins.

To understand the functional consequence of EEA1 monoubiquitination, we took advantage of the observation that in-frame fusion of ubiquitin to a protein often mimics endogenously generated mono-ubiquitinated protein [24,25]. We substituted the C-terminal glycine in ubiquitin to valine to avoid cleavage of ubiquitin by cellular deubiquitinases and then fused the coding sequence of UbG76V to the 5′-end of the EEA1 gene. This construct allowed the expression of a constitutively 'ubiquitinated' EEA1 variant (Ub-EEA1), carrying just a single ubiquitin moiety at the N-terminus. We expressed EEA1 or Ub-EEA1 in COS7 cells and stained the cells with an EEA1 antibody to visualize the endosomes. In untransfected cells, the EEA1 antibody stains clusters of small vesicles in a perinuclear region. Since EEA1 normally functions as a tether that ‘primes’ endosomes for fusion, overexpression of wild type EEA1 induced endosome clustering and fusion, resulting in enlarged endosome vesicles (Figure 3A). Intriguingly, approximately 60% of cells expressing Ub-EEA1 contained giant vacuole-like EEA1-positive membrane structures in proximity to the nucleus (Figures 3A, B). Co-expressing the early endosome marker Rab5-GFP showed that Ub-EEA1 and Rab5 were co-localized in this structure (Additional file 1: Figure S1), confirming that this structure originated from early endosome vesicles, likely due to uncontrolled fusion of early endosomes. Under electron microscopy, Ub-EEA1-expressing cells often contained large vacuole-like structures resembling late endosome/lysosome (Figure 3C). Since immunoblotting showed that Ub-EEA1 was expressed at a much lower level than EEA1 (Figure 3D), Ub-EEA1 appeared much more active than unmodified EEA1 in inducing endosome fusion, causing enlarged and seemingly over-matured endosomes.

To see if the enlarged endosome phenotype depends on the interplays between Ub-EEA1 and an unknown ubiquitin receptor in cells, we substituted Ile44 in Ub-EEA1 with alanine. This isoleucine residue is required for ubiquitin recognition by all forms of ubiquitin binding domains in cells. Thus, if an ubiquitin binding effector is required for Ub-EEA1 to induce enlarged endosomes, the mutation should abolish the phenotype. Indeed, even though UbI44A-EEA1 was expressed at a higher level than Ub-EEA1, as demonstrated by immunoblotting (Figure 3D), cells expressing this mutant only displayed a small increase in size of endosomes, comparable to cells expressing EEA1 (Figure 3A). Similar observations were made using a set of EEA1 constructs that carried a FLAG tag in addition to the ubiquitin fusion in COS7 cells and in another mammalian cell line, the HEK293 cells (Additional file 1: Figure S2).

**Figure 4:**
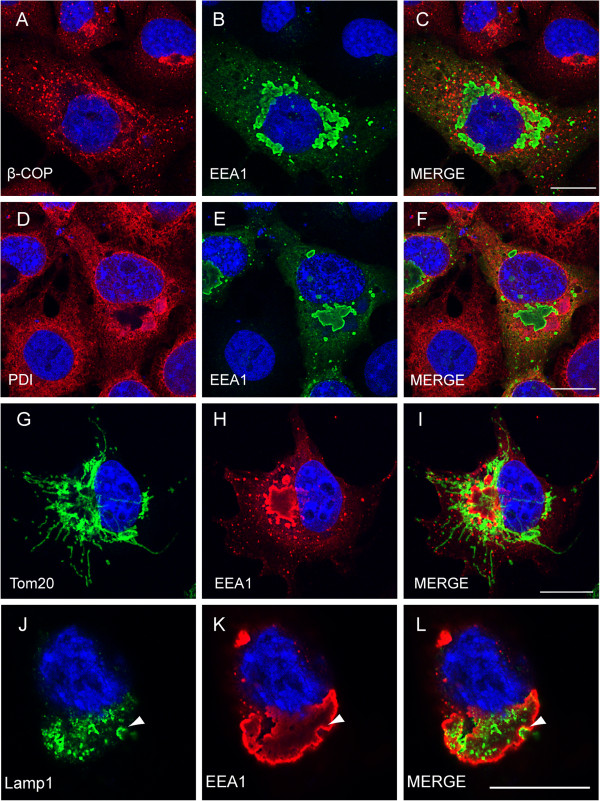
**Expression of Ub-EEA1 alters Golgi morphology and induces fusion of lysosome with endosome.** COS7 cells expressing Ub-EEA1 were fixed and immunostained with EEA1 antibody (**B**, **E**, **H**, **K**) together with antibodies against β-COP (**A**), PDI (**D**), Tom20 (**G**), or Lamp1 (**J**). Cells were also stained with DAPI in blue to reveal the nuclei. Merged two-color images are shown in **C**, **F**, **I**, **L**. Scale bars correspond to 20 μm.

We further characterized the giant endosome structures generated by Ub-EEA1 using antibodies that label a variety of subcellular organelles. The results showed that the Ub-EEA1 positive endosomes did not co-localize with the Golgi marker β-COP. Interestingly, the morphology of the Golgi in cells expressing Ub-EEA1 was dramatically altered compared to control cells. Instead of forming a stack in a perinuclear region, β-COP positive Golgi vesicles are completely dispersed throughout the cell, suggesting a connection between the endocytic pathway and Golgi morphology (Figure 4A-C). By contrast, the ER and mitochondrial morphology were largely unaffected, and Ub-EEA1 did not colocalize with either ER or mitochondrial markers (Figure 4D-I). Intriguingly, immunostaining with a LAMP1 antibody that labels lysosomes showed that many lysosome vesicles were docked on the large endocytic structures formed upon Ub-EEA1 expression (Figure 4J-L). These results suggest that Ub-EEA1 disrupts endocytic homeostasis at least in part by inducing uncontrolled fusion of endosomes that can fuse further with the lysosomes. The results also suggest that endocytic membrane homeostasis is critical for maintaining normal Golgi stack in cells,

### EEA1-Ubiquitin expression blocks internalization of transferrin

It was known from previous studies that constitutive activation of Rab5 induces uncontrolled fusion of early endosomes, resulting in giant endosomes [26]. However, endosomes generated by Rab5 activation (e.g. expression of the constitutive activated Rab5) are morphologically distinct from those produced by Ub-EEA1. The former generates enlarged endosomes that are mostly spherical whereas as the endosome sheets formed by Ub-EEA1 are often irregular in shapes. In addition, the endosome structures generated by Ub-EEA1 are also generally larger than those by Rab5 Q79L expression. These differences suggest that uncontrolled fusion may not be the only contributor that forms the abnormal endocytic structures in Ub-EEA1 expressing cells.

**Figure 5:**
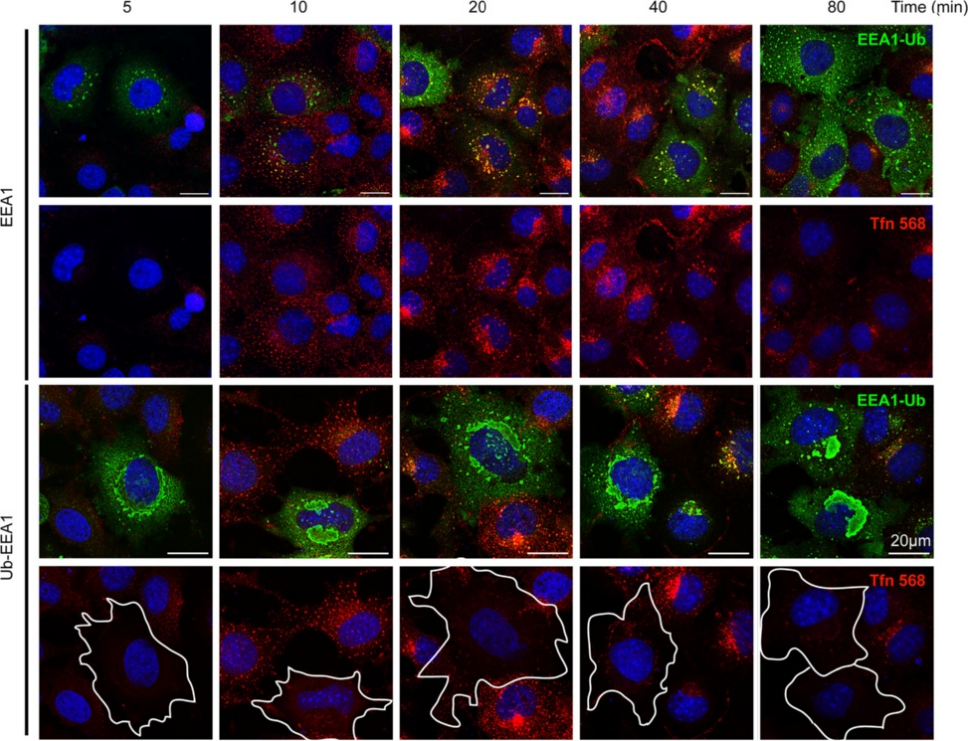
**Expression of Ub-EEA1 inhibits transferrin uptake.** COS7 cells transfected with EEA1- or Ub-EEA1-expressing plasmids were treated with Texas red-labeled transferrin on ice. After removal of unbound transferrin, cells were incubated in a transferrin-free medium at 37°C for the indicated time points. Cells were fixed and stained with anti-EEA1 antibody (green) and DAPI (blue). Note that the highlighted Ub-EEA1-expressing cells fail to take up transferrin compared to the surrounding untransfected cells.

We examined whether Ub-EEA1-expressing cells had other endocytic trafficking defects. We first analyzed the internalization of Texas red-labeled transferrin in cells expressing either EEA1 or Ub-EEA1. Following incubation of the cells with labeled-transferrin on ice, we removed unbound transferrin by washing and then incubated the cells at 37°C in a transferrin free medium for different time points. In EEA1-expressing cells, transferrin was internalized and accumulated in EEA1 positive endosomes over time (Figure 5). In general, cells internalizing transferrin gradually lost the transferrin signal as a result of an endocytic recycling process. Interestingly, cells expressing Ub-EEA1 failed to take up transferrin (Figure 5). We questioned whether enlargement of endosomes in general can block transferrin uptake. We therefore performed the transferrin uptake experiment using cells expressing Rab5 Q79L. Despite the presence of a large number of enlarged endosomes, these cells effectively internalized Texas red-labeled transferrin (Additional file 1: Figure S3), consistent with a previous report [26]. These results further underscore the phenotypic difference between cells containing activated Rab5 and those expressing Ub-EEA1.

### Ubiquitin-EEA1 traps a cell surface receptor at endosomes

One possible explanation for the enlarged endosome phenotype associated with Ub-EEA1 is that activation of EEA1 by ubiquitination may not only induce endosome fusion, but also block an endosome fission process required for recycling of certain membrane receptors such as the transferrin receptor to the plasma membrane. This could lead to a depletion of transferrin receptor from the cell surface, resulting in abnormal transferrin uptake. This model also explains why the endosomes in Ub-EEA1 cells are even larger than those in Rab5 Q79L expressing cells, as the endosome enlargement induced by Rab5 activation may be solely due to increased vesicle fusion.

**Figure 6:**
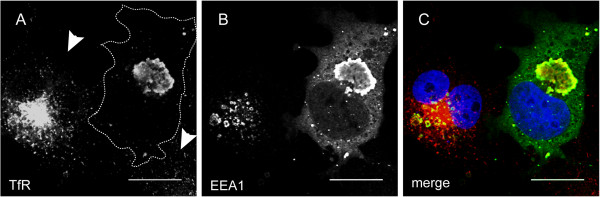
**Expression of Ub-EEA1 blocks the recycling of the transferrin receptor.** COS7 cells transfected with the Ub-EEA1-expressing plasmid were fixed and immunostained with an antibody against transferrin receptor (TfR) (**A**, red channel in C) and anti-EEA1 antibody (**B**, green channel in C). Cells were also stained with DAPI in blue. Arrow heads label two untransfected cells that contain many transferrin receptor molecules in recycling vesicles. By contrast, the circled Ub-EEA1-expressing cells contain few TfR positive recycling vesicles. The scale bars correspond to 20 μm.

To test this hypothesis, we stained cells with an antibody that recognizes the transferrin receptor. In wild type cells, transferrin receptor was mostly localized in endosomal vesicles concentrated in a perinuclear region, but small vesicles localized to peripheral regions of the cells were also frequently observed. These vesicles represented recycling endosomes, which are responsible for re-distributing internalized transferrin receptor to the plasma member. Only a few transferrin receptor molecules were detected on the plasma membrane, suggesting that it was transiently localized to the cell surface. In Ub-EEA1 expressing cells, transferrin receptor was almost entirely trapped in the enlarged endocytic structures. Almost no transferrin receptor positive recycling endosome vesicles could be detected (Figure 6). This observation supports our model that overactivation of EEA1 via ubiquitin conjugation not only induces endosome fusion, but also blocks endosome recycling.

## Discussion

We showed previously that the p97 ATPase can regulate the oligomeric state of EEA1 to control endosome morphology and trafficking kinetics [15]. Given that p97 substrates are often polyubiquitinated [27,28], we investigate whether EEA1 is ubiquitinated in cells. To our surprise, we find that EEA1 preferentially undergoes monoubiquitination in cells. In many p97-mediated biological processes studied to date, the functional consequence of p97 action is to separate ubiquitinated substrates from large immobile structures, therefore facilitating their degradation by the proteasome. This has been best demonstrated for the degradation of misfolded proteins of the endoplasmic reticulum (ER) via the ER-associated degradation (ERAD) pathway [29,30]. Because EEA1 is mostly modified with single ubiquitin moieties, a functional interplay between ubiquitinated EEA1 and p97, if exists, may not be directly involved in p97-dependent modulation of the EEA1 oligomeric state. In support of this notion, we find that unlike many p97 substrates, inhibition of p97 does not significantly increase the levels of ubiquitinated EEA1. In addition, p97 co-precipitates with both modified and unmodified EEA1 with apparently similar affinities (data not shown). Because the activity of the ubiquitin-EEA1 fusion protein is dependent on Ile44 of ubiquitin, it is possible that monoubiquitination of EEA1 may recruit an unknown ubiquitin receptor, which may in turn interfere with p97-dependent disassembly of EEA1. Alternatively, monoubiquitination of EEA1 may compete with a parallel polyubiquitination event essential for disassembly of an EEA1 oligomer complex by p97.

Protein monoubiquitination can occur on many substrates in cells via a variety of mechanisms. One well-known method of monoubiquitination is via an E3 independent mechanism. In this case, monoubiquitination occurs when a substrate make direct contact with a ubiquitin charged E2 enzyme. The substrate can either directly interact with the E2 or use a ubiquitin binding motif to associate with the ubiquitin molecule at the E2 active site [22]. We show that ubiquitination of EEA1 is apparently due to an intrinsic affinity between EEA1 and E2 ubiquitin conjugating enzymes. Our in vitro study suggests that EEA1 can be ubiquitinated by several E2 enzymes in the absence of any E3 ubiquitin ligases. The functional interplay between EEA1 and E2 conjugating enzymes are corroborated by our protein interaction study, which demonstrate a direct interaction between EEA1 and Ube2A. Although Ube2A appears to be the most efficient E2 that ubiquitinates EEA1 in vitro, depletion of Ube2A and Ube2B simultaneously using siRNA-mediated gene silencing does not significantly affect the levels of ubiquitinated EEA1 in cells. It is possible that other E2 enzymes may substitute Ube2A and Ube2B in their absence. Alternatively, Ube2A and Ube2B may not be the major E2 for EEA1 ubiquitination given its preferred nuclear localization. Further experiments would be required to identify the E2s responsible for EEA1 ubiquitination in cells. In addition, our current data cannot exclude that cells may use an E3-dependent mechanism to mono-ubiquitinate EEA1. Despite these uncertainties, the intrinsic affinity between EEA1 and E2 enzymes as demonstrate in this study clearly indicates the likelihood that an EEA1 molecule in cells can just receive ubiquitin directly from one or more E2s, which are abundant in cells.

One unexpected observation of this study is that expression of a Ub-EEA1 fusion protein mimicking ubiquitinated EEA1 induces giant endocytic structures distinct from those generated by over-activating Rab5. Constitutively activated Rab5 recruits wild type EEA1 to the endosome membrane, which results in enlarged endosomes that are sphere-shaped. By contrast, the endosomes generated by Ub-EEA1 expression are larger in size and more irregular in appearance. Interestingly, despite being driven by the same promoter, EEA1 and ubiquitin-EEA1 are expressed in cells at different levels. Ubiquitin moiety fused to EEA1 seems to impart instability, resulting in a much lower level of expression. Ub-EEA1 bound to the endosome membrane may be constantly turned over by the lysosomes that are associated with the abnormal endocytic structures. Although Ub-EEA1 is expressed at a lower level than EEA1, the endosomes generated by Ub-EEA1 are much larger than those formed upon EEA1 expression. Thus, ubiquitinated EEA1 seems to represent an over-activated form that drives the fusion and maturation of those vesicles associated with it. Since the activity of Ub-EEA1 is dependent on isoleucine 44 in the attached ubiquitin, it is reasonable to presume that its activity is dependent on a cellular ubiquitin receptor capable of recognizing both ubiquitin and EEA1. Regulation of the size of intracellular vesicles by monoubiquitination of a fusion factor has not been reported previously. However, a recent study demonstrated that monoubiquitination of the COPII component SEC31 can increase the size of COPII-coated secretory vesicles [31].

The ubiquitinated EEA1 only constitutes a small fraction of the entire pool of EEA1 at the steady state, in line with other substrates undergoing ubiquitination [31]. This observation suggest that ubiquitinated EEA1 may act catalytically to induce endosome fusion. Moreover, ubiquitination of endogenous EEA1 may be normally antagonized in cells by cellular deubiquitinases, a family of ubiquitin isopeptidases that remove ubiquitin conjugates from substrate proteins [32]. By limiting ubiquitinated EEA1, cells may avoid overflow of endocytic vesicles to the lysosomes. Several deubiquitinases have been found to be associated with the endocytic membrane system including USP8 and AMSH [33-35]. However, siRNA-mediated depletion of these proteins does not affect the levels of ubiquitinated EEA1 in cells (data not shown). Identification of deubiquitinases for EEA1 may further reveal the mechanism and biological significance of EEA1 ubiquitination in the context of endocytic trafficking.

## Conclusions

In summary, our study establishes EEA1 as a ubiquitin-regulated factor that may be modified via an E3 independent ubiquitin conjugation mechanism in cells. In addition, ubiquitination may significantly alter EEA1 activity to influence endocytosis.

## Methods

### Cell culture, plasmid preparation, transfection

COS7 and HEK293 cells were purchased from ATCC. Full length human EEA1 was purchased (OriGene). EEA1 deletion mutants were made by introducing SgfI or MluI sites at desired positions using PCR based site-directed mutagenesis. Restriction digestion using MluI or SgfI and religation yielded desired deletion mutants. To make plasmid expressing the EEA1-ubiquitin fusion protein, the ubiquitin open reading frame was amplified with primers carrying mutation to generate a ubiquitin G76V variant. The primers also included EEA1 sequences flanking the ubiquitin sequences. The resulting PCR product was used as primers for subsequent site-directed insertional mutagenesis using EEA1 or EEA1-FLAG expressing plasmid as templates to yield in frame N-terminal Ub G76V fusion to EEA1. Site directed mutagenesis was performed using a kit from Strategen following manufacture’s protocol. TransIT-293 (Mirus) and FuGENE 6 (Roche) were used for plasmid transfection in HEK293 and COS7 cells, respectively.

### Antibodies, proteins, and chemicals

Rabbit anti-EEA1 (Cell Signaling), rabbit anti-FLAG (Sigma); mouse anti-EEA1 (BD Biosciences); rabbit anti-β-COP (Thermo Scientific, IL), rabbit PDI (Enzo life sciences, NY), mouse Tom20 (clone F10, Santa Cruz Biotechnology, CA) and mouse Lamp1 (clone H5G11, Santa Cruz Biotechnology, CA) were used. Transferrin-568, goat anti-mouse or goat anti-rabbit conjugated to Alexa-568 and Alexa-488 were from Invitrogen. Goat anti-mouse conjugated to infrared dye IR680 and goat anti-rabbit conjugated to IR800 were from Rockland (Rockville, MD). GST-E1 and ubiquitin conjugating enzyme used in this study were purchased from BostonBiochem. Purification of Ube2g2 was described previously [36].

### Purification of EEA1

To purified EEA1, HEK293 cells were transfected with a plasmid expressing FLAG-tagged full length EEA1. Cells were harvested 72 hours post transfection in a buffer containing 1% DeoxyBig CHAP, 30 mM Tris. HCl pH 7.4, 150 mM potassium acetate, 4 mM magnesium acetate and a protease inhibitor cocktail. The cleared cell extract was incubated with FLAG-agarose beads (Sigma) and the bound materials were extensively washed with a buffer containing 0.1% DeoxyBig CHAP, 30 mM Tris. HCl pH 7.4, 150 mM potassium acetate, 4 mM magnesium acetate. The bound materials were eluted using 0.2 mg/ml 3X FLAG peptide (Sigma) in 25 mM Hepes, pH 7.2, 115 mM potassium acetate, 5 mM sodium acetate, 2.5 mM magnesium acetate.

### In vitro ubiquitination

To ubiquitinate EEA1 using PNS, HEK293 cells treated with a hypotonic low salt buffer (50 mM Hepes, 7.3, 10 mM potassium chloride, 2 mM magnesium chloride, 0.5 mM DTT, and a protease inhibitor cocktail) were homogenized with a Dounce-homogenizer. After centrifugation at 1000 g for 5 min at 4°C, the postnuclear supernatant (PNS) fraction was collected and used in an in vitro ubiquitination reaction containing 25 μg HA-tagged ubiquitin, 1 μg ubiquitin aldehyde, 0.2 μg GST-E1, an ATP regenerating system, ~1 mg PNS in a 120 μl volume. After incubation at 37°C, aliquots taken at different time points were lysed in the NP40 lysis buffer (50 mM Tris–HCl pH 7.4, 150 mM sodium chloride, 2 mM magnesium chloride, 0.5% NP40, and a protease inhibitor cocktail). EEA1 immunoprecipitated with FLAG beads was analyzed by immunoblotting. In vitro ubiquitination with purified components were performed in 20 μl reaction buffer (25 mM Tris HCl, pH 7.4, 2 mM magnesium/ATP, and 0.1 mM DTT) containing 40 ng GST-E1, 4 μl HA-ubiquitin, ~0.5 μg EEA1-FLAG, an ATP regenerating system, and 1 μl indicated E2 conjugating enzymes. The reaction was stopped by addition of the Lamili buffer and analyzed by immunoblotting.

### Surface plasmon resonance interaction analysis

Surface plasmon resonance interaction experiments were carried out at 25°C on a Biacore 2000 system (GE Healthcare). Purified EEA1-FLAG was covalently attached to a carboxymethyl dextran-coated gold surface (CM5 Chip; GE Healthcare) using an amide couple kit following the manufacturer’s suggested protocol. Briefly, the carboxymethyl group of dextran was activated with N-ethyl-N’-(3-dimethylaminopropyl) carbodiimide (EDC) and N-hydroxysuccinimide (NHS). EEA1-FLAG in a buffer containing 10 mM sodium acetate (pH 4.5) was injected at a flow rate of 10 μL/min with 2-min contact time, resulting in the immobilization of EEA1-FLAG at a level of ~2,000 RU. Any remaining reactive sites in the flow cell were blocked by ethanolamine. The E2 interactions were monitored at a flow rate of 20 μL/min with the E2 concentration ranging from 2.5 to 160 μM. Analysts were prepared in a buffer containing 10 mM Hepes (pH 7.4), 150 mM NaCl, 3 mM EDTA, 0.005% (vol/vol) surfactant P20. Regeneration was achieved by injecting a buffer containing 2 M NaCl. SPR signal was normalized by using a reference flow cell containing no EEA1. For each E2 variant, at least 2 measurements were made.

### Immunostaining, confocal microscopy and electron microscope analyses

For EEA1 staining, cells plated on coverslips for 24 h were fixed in 2% formaldehyde in PBS for 10 min, rinsed in PBS and blocked for 15 min in PBS containing 10% FBS. Post-blocking, the cells were rinsed and incubated with mouse or rabbit anti-EEA1 primary antibodies (1:100 dilution) in PBS containing 10% FBS and 0.2% saponin. For p97 staining, cells were fixed in 4% paraformaldehyde containing 250 mM sucrose, permeabilized and stained with mouse anti-p97 antibody in PBS containing 0.1% Igepal CA-630 and detected with secondary goat anti-mouse or goat anti-rabbit secondary antibodies conjugated to Alexa fluro-568 or 488. To track endocytic cargoes, cells were washed and starved for 30 min in serum-free DMEM containing 0.5% BSA followed by 15 min incubation with labeled Transferrin (5 μg/ml) on ice. The cells were washed twice with PBS and the bound labeled cargoes were allowed to internalize for different time points by incubating the cells at 37°C. All images were obtained using a 510 LSM Confocal microscope (Zeiss) with a 10× Plan Apo objective and processed using Adobe Photoshop 7.0. For electron microscope analyses, COS7 cells were treated with either DMSO or 10 μM EerI for 3 h. Cells were washed with PBS and then fixed with 2% glutaraldehyde in 0.1 M cacodylate buffer at room temperature for 1 h prior to electron microscope analysis.

## Competing interests

The authors declare that they have no competing interests.

## Authors’ contributions

HR and YY designed the study and analyzed the data. HR carried out all the biochemical and fluorescence imaging studies. GZ performed the EM experiments. YY wrote the paper. All authors read and approved the final manuscript.

## Supplementary Material

Additional file 1: Figure S1 EEA1 is co-localized with Rab5. **Figure S2**. Ub-EEA1 expression generates enlarged endosomes. **Figure S3**. Rab5 Q79L expression enlarges early endosomes, but does not affect transferrin trafficking. Click here for file
