# Supplementary material for: Monoubiquitination of EEA1 regulates endosome fusion and trafficking
Source: Cell Biosci. 2013 May 23;3:24. doi: 10.1186/2045-3701-3-24 (PMC3673817; doi:10.1186/2045-3701-3-24)
Supplement: Additional file 1: Figure S1 — EEA1 is co-localized with Rab5. Figure S2. Ub-EEA1 expression generates enlarged endosomes. Figure S3. Rab5 Q79L expression enlarges early endosomes, but does not affect transferrin trafficking. [file 2045-3701-3-24-S1.doc]

**Supplemental information**

Figure 1


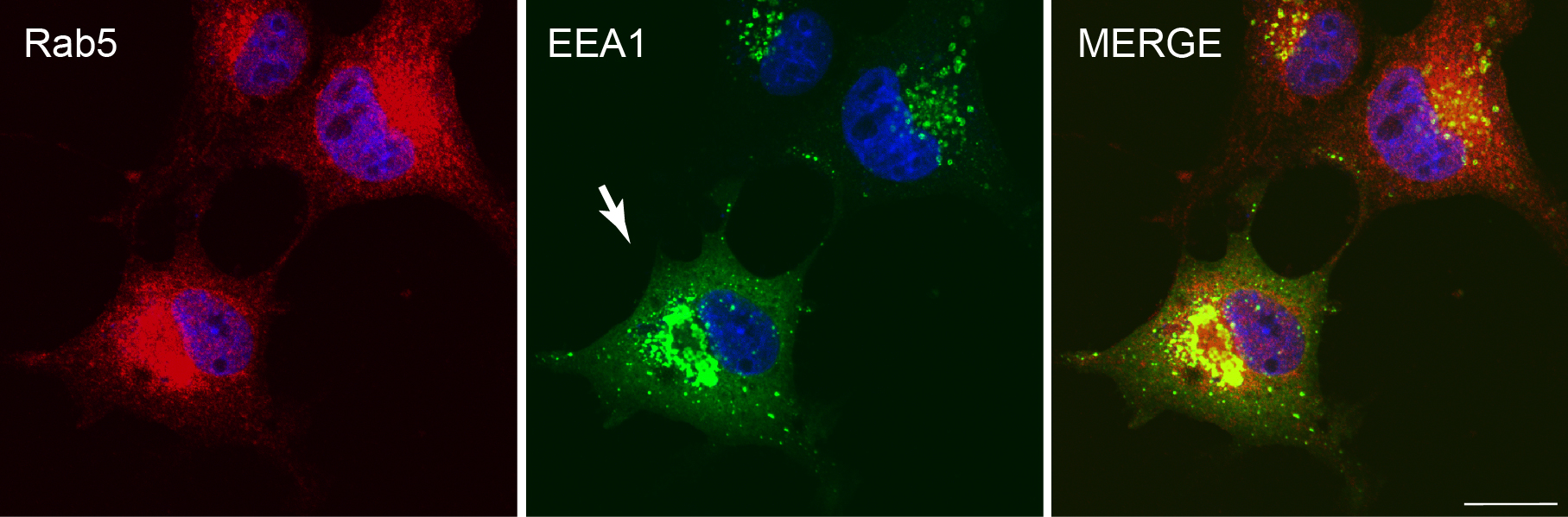


**Figure 1 EEA1 is co-localized with Rab5**

COS7 cells transfected with a Ub-EEA1-expressing plasmid were stained with antibodies against Rab5 (red) and EEA1 (green). The cells were also stained with DAPI to show the nuclei. The arrow indicated a Ub-EEA1-expressing cells. The scale bars correspond to 20μm.

Figure 2


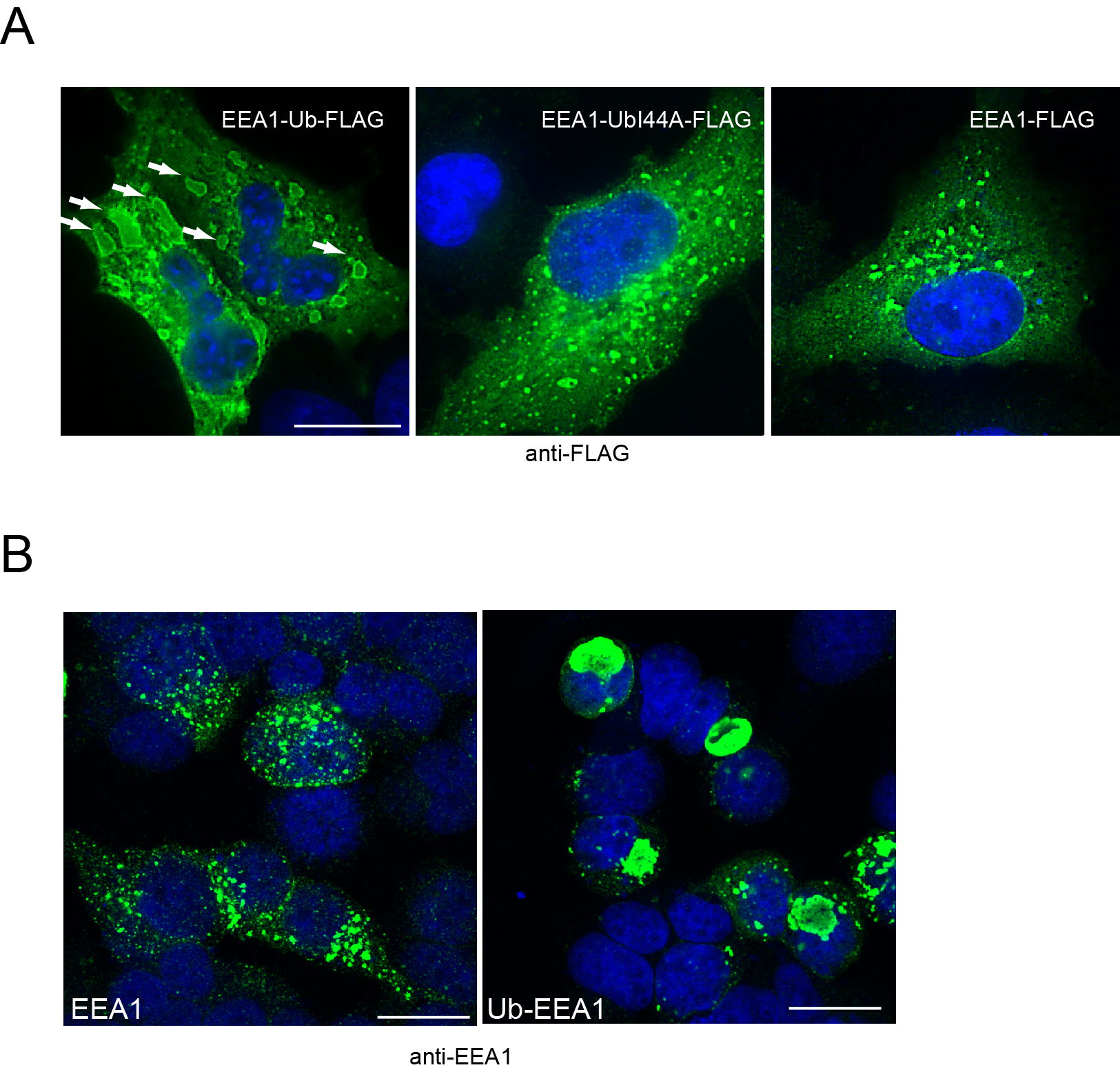


**Figure 2 Ub-EEA1 expression generates enlarged endosomes**

A. COS7 cells transfected with plasmids expressing the indicated proteins were stained with FLAG antibodies (green) and DAPI (blue). Arrows indicate enlarged EEA1-positive endosomes. B. As in A, except that HEK293 cells transfected with either EEA1 or Ub-EEA1 were used. The scale bars correspond to 20μm.

Figure 3


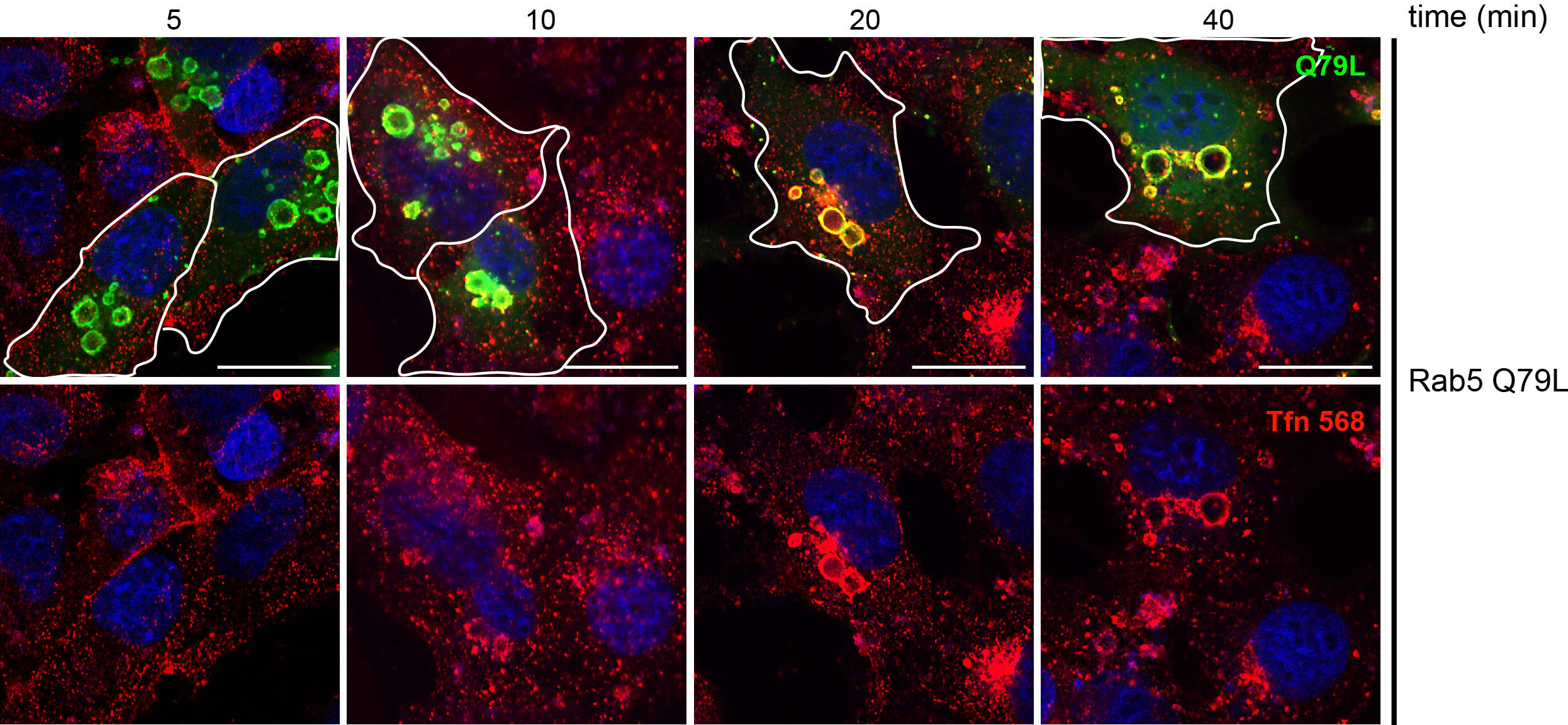


**Figure 3 Rab5 Q79L expression enlarges early endosomes, but does not affect transferrin trafficking**

COS7 cells transfected with Rab5 Q79L-expressing plasmids were treated with Texas red-labeled transferrin on ice. After removal of unbound transferrin, cells were incubated in a transferrin-free medium at 37 ºC for the indicated time points. Cells were fixed and stained with anti-EEA1 antibody (green) and DAPI (blue). The scale bars correspond to 20μm.
